# Triterpenoids from *Psidium guajava* with Biocidal Activity

**DOI:** 10.4103/0250-474X.73936

**Published:** 2010

**Authors:** P. Ghosh, A. Mandal, P. Chakraborty, M. G. Rasul, Madhumita Chakraborty, A. Saha

**Affiliations:** Natural Product and Polymer Chemistry Laboratory, Department of Chemistry, University of North Bengal, Darjeeling – 734 013, India; 1 Department of Botany, University of North Bengal, Darjeeling – 734 013, India

**Keywords:** Betulinic acid, lupeol, minimum inhibitory concentration, *myrtaceae*, *Psidium guajava*, triterpenoid

## Abstract

In continuation of our studies on the phytochemical investigation of medicinal plants available in the foothills of Darjeeling and Teri, we report herein the isolation of two triterpenoids betulinic acid and lupeol from the leaf extract of *Psidium guajava* and their potential antimicrobial and phytotoxic activities. All the structures of the isolated compounds were confirmed by spectral (IR, NMR) analysis and by comparison with the literature reports.

The Himalayan region of Darjeeling and Terai are rich in bio diversity with plants having pronounced medicinal activities as evidenced by recent literature reports[1–3] as well as by the tribal medicinal practice in this region. Plants of the family *Myrtaceae* are extensively used in indigenous medicine from prehistoric ages. *Psidium guajava* is an important representative of this family. Present day reports about *P. guajava* are attracting because of their highly encouraging biological activities[3–11]. Different parts of these plants are used in the traditional system of medicine for the treatment of various human ailments such as ulcers, bronchitis, eye sores, bowels, diarrhoes and cholera[3–6]. It is reported in the literature that the leaf extract of *P. guajava* has antitussive, antibacterial, hemostatic, antioxidant and narcotic properties[7–10]. Recently Abreu *et al*, have reported that guava extract can alter the labelling of blood with technetium-99m[11].

In view of the attributed medicinal properties and in an ongoing search for bioactive triterpenoids from plants of *Myrtaceae* available in Darjeeling foothills, the toluene extract of leaves of *P. guajava* was selected for further investigation. The leaf extract of *P. guajava* was found to contain two new triterpenoids (1 and 2) along with earlier reported guajanoic acid (3)[6], β-sitosterol (4), uvaol (5), ursolic acid (6) and oleanolic acid (7) (fig. 1). Compounds 1 and 2 have been characterized as betulinic acid and lupeol respectively. This is the first report of the isolation of these two triterpenoids from the leaf extract of *P. guajava* available plenty in the foothills of Darjeeling. In addition to that preliminary studies towards the antimicrobial and phytotoxic activities of these two compounds, which have not yet reported so far from this source, have also been carried out against some fungal and bacterial pathogens.

**Fig. 1:**
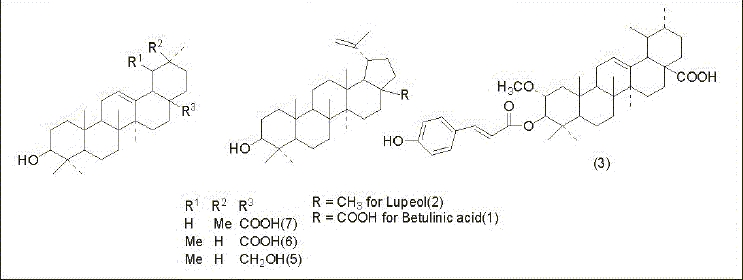
Isolated triterpenoids from Psidium guajava

All the melting points were determined by open capillary method and are uncorrected. The NMR spectra were recorded in CDCl_3_ solutions at ambient temperature on a Bruker Avance 300 MHz-FT NMR spectrometer using 5 mm BBO probe. The chemical shift δ are given in ppm related to tetra methyl silane (TMS) as internal standard. The coupling constant (*J*) are reported in Hz. The IR spectra were recorded in Shimadzu FT-IR spectrophotometer in KBr discs.

Fresh leaves of *P. guajava* were collected in bulk from young mature plants at the Sukna belt of Darjeeling foothills during early summer, washed, shade dried and milled into coarse powder by a mechanical grinder. The prepared powdered leaves were then used for further studies. The powdered plant material was extracted with toluene using Soxhlet apparatus for 72 h. The solvents were then removed under reduced pressure and a sticky brown residue was obtained. This residue was then purified by column chromatography using silica gel (60-120) mesh and suitable proportions of petroleum ether and ethyl acetate were used as the eluent.

In this present work the *in vitro* antifungal antibacterial activities and the phytotoxicity of the two isolated triterpenoids have been studied. Five different fungal pathogens namely, *Calletotricheme camellie, Fussarium equisitae, Alterneria alternate, Curvularia eragrostidies, Colletrichum Gleosproides* were used for the antifungal study. For antibacterial study *Escherichia Coli, Bacillus Subtilis, Staphylococcus aureus, Enterobactor* were used as bacterial pathogen. Suitable strains of these organisms were procured from the microbiology laboratory of our institute. MICs (minimum inhibitory concentration) of the triterpenoids against bacterial and fungal pathogens are reported in Tables 1 and 2, respectively. DMSO (dimethyl sulfoxide) was used as solvent to prepare different concentrations of the triterpenoids. Solvent control (DMSO) was also maintained throughout the experiment. All experiments were performed in petridishes and were incubated at 37° for 48 h.

The bacterial growth was confirmed by a change of yellow to purple colour. Bacterial nutrient media was prepared by using agar, beef extract and bacto peptone in distilled water and the pH of the solution (6.8-7.0) was adjusted. Culture media for fungal strains were prepared by mixing in suitable proportions of potato extract, dextrose and agar powder. All glass apparatus, culture media were autoclaved before use. The whole process was carried out in inoculation chamber. Additionally slide germination method was also used for determination of antifungal activity[12] (Table 3). The antifungal activities between these compounds and streptomycin and antibacterial activity with ampicillin, a β-lactam antibiotic were compared.

**TABLE 1:** MIC OF 1 AND 2 AGAINST DIFFERENT BACTERIA

Compounds	MIC in μg/ml against different strains of bacteria			
	EC	BS	SA	EB
1	150	<100	100	100
2	200	100	200	100
Ampicillin	128	64	64	128

BS- *Bacillus. subtilis*, EC- *Escherichia coli*, SA- *Staphylococcus aureus*, EB-*Enterobactor*, MIC- Minimum inhibitory concentration

**TABLE 2:** MIC OF 1 AND 2 AGAINST DIFFERENT FUNGI

Compounds	MIC in μg/ml against different fungi			
	CG	FE	CE	AA	CC
1	<5	2.5	10	5	<5
2	10	5	10	5	5
Streptomycin	1.25	2.5	<2.5	2.5	2.5

CG- *Colletrichum gleosproides*, FE- *Fussarium equisitae*, CE- Curvularia *eragrostidies*, AA- *Alterneria alternate*, CC- *Calletotricheme camellie*.

**TABLE 3:** ANTIFUNGAL PROPERTIES OF 1 AND 2 BASED ON SPORE GERMINATION BIOASSAY

FungalPathogen	Betulinic acid			Lupeol		
	PG^a^	PI	AL^b^(μm)	PG^a^	PI	AL^b^(μm)
CC	00	100	00	0.5	95	4.5
FE	00	100	00	00	100	00
AA	00	100	00	00	100	00
CG	00	100	00	00	100	00
CE	00	95	6.0	10	95	9.0

CG- *Colletrichum Gleosproides*, FE- *Fussarium equisitae*, CE- *Curvularia eragrostidies*, AA- *Alterneria alternate*, CC- *Calletotricheme camellie*. PG-Percent germination, PI-Percent Inhibition, AL-Average germ tube length,

a Based on 200 spores,

b Based on 25 germ tubes. All data were taken after 48 h of incubation.

For studying the inhibitory effect[12] of the two triterpenoids against test fungal pathogens following slide germination method, the spores of the pathogens were allowed to germinate in presence of the prepared and the 50% ethanol extracts. Compound solution was placed on the centre of the grease free microscope slide. In control the corresponding solvent, either sterile distilled water or 50% ethanol was placed. Thirty microlitre spore suspension prepared from ten days culture of the fungal pathogens were added to the spots in both experimental and control slides. In case of 50% ethanol extract, spore suspension was added after ethanol was evaporated. Three experimental slides were taken for each compound. The slides were then incubated at 28° in a humid chamber. Two small glass rods (60 mm in length) were placed in a 90 mm Petri dish and a slide was placed on the rods in a uniformly balanced position. Then the Petri dish was filled with sterile distilled water so that the bottom of the slide remained just above the water surface. The petridish was then covered and incubated at 28°. Following 48 h of incubation, the slides were stained with lacto phenol-cotton blue mixture and observed in each slide for germination. Numbers of aspersoria formed were also observed and lengths of 50 germ tubes were measured. The entire experiment was repeated thrice.

Seeds of rice (*Oriza sativa*), wheat (*Triticum aestirium*), and pea (*Pisum sativum*) were collected from local market. The assay seeds were shorted for uniformity of size and all damaged seeds were discarded. Before the bioassay seeds were washed with tap water and the surface were sterilized using NaCl (10% v/v) for 10 min followed by several washes in sterile distilled water. For testing phytotoxicity dehydrated ethanol was used as control. Bioassays were carried out using petridishes (90 mm diameter) containing a sheet of Whatman 1 filter paper as support. Test solutions (5 ml) was added to the filter paper in the petridish and dried completely *in vacuo* at 40°. Five seeds from each category were placed on the filter paper and incubated for 7 days at 25° in the dark. The effects of the pure compounds were determined by measuring the elongation of roots and averaged for each concentration.

Compound (1) was isolated as white crystals (CHCl_3_+MeOH) of m.p. 299-301°. IR spectrum has exhibited hydroxyl at v_max_ 3610, 1020 cm^-1^ and exomythylene at v_max_ 3060, 1630, 880 cm^-1^. The^1^ H NMR spectrum revealed signals for five tertiary methyls δ_H_ 0.65, 0.75, 0.90, 0.96 and 0.98, a vinyl methyl δ_H_=1.97 broad d, J= 0.5 Hz), a secondary carbinol δ_H_=3.16 dd, J= 9.5 and 6.0 Hz) and δ_H_= 2.95 (ddd, J= 9.0, 6.0 and 0.5 Hz) an exomethylene group δ_H_=·4.55 (1H, d, J= 0.4 Hz) and δ_H_= 4.65(1H, d, J= 0.4 Hz). These data indicated a pentacyclic triterpenoid of betulinic acid, confirmed by comparison with already published data[13–16]. The ^13^C NMR spectrum of (1) showed six methyl group at δ_C_ 27.9 (C-23), 15.4 (C-24), 16.2 (C-25), 16.3 (C-26), 14.6 (C-27), 19.6 (C-30) and exomethylene group at δ_C_ 150.0 (C-30), 108.8 (C-29) and a secondary carbon bearing hydroxyl at δc 79.0 (C-3) and a carboxyl group at δ_C_= 180.6 (C-28) in addition to ten methylene, five methine and five quaternary carbons. These data were identical to those reported for betulinic acid[13–16].

Lupeol (2) was isolated as white crystals from CHCl_3_+MeOH mixture and gave m.p. 210-212° [α]_D_= +30.4 (conc. 0.58 in CHCl_3_). Its IR spectrum exhibited hydroxyl at v_max_ 3610, 1020 cm^-1^ and exomethylene at v_max_ 3070, 1640, 887 cm^-1^ absorption. The^1^ H NMR exhibited six tertiary methyl signals at δ_H_ 0.75, 0.77, 0.80, 0.92, 0.94 and 1.02, a vinyl methyl group at δ_H·_ 1.66 (broad d J= 0.5 Hz)], a secondary carbinol group at δ_H_ 3.20 (dd, J= 9.6 and 6.2 Hz) and an exomethylene group at δ_H_ 4.58 (1H, d, J= 0.4 Hz) and δ_H_= 4.65 (1H dq, J= 0.4 and 0.5 Hz) typical of pentacyclic triterpenoid[15,16] of lupeol (2). The structural assignment of (2) was further substantiated by its^13^ C NMR spectrum which showed seven methyl groups at δ_C_ 28.0 (C-23), 19.3 (C-30), 18.0 (C- 28), 16.1 (C-25), 15.9 (C-26), 15.4 (C-24), 14.5 (C-27), an exomethylene group at δ_C_ 150.8 (C-20), 109.3 (C-29) and a secondary hydroxyl bearing carbon at δ_C_ 78.9 (C-3) in addition to ten methylene, five methine and five quaternary carbons. Shielding of C- 23 methyl of (2) could be due to the influence of the adjacent C-3 hydroxyl group. These data were in close agreement with those reported for lupeol (2)[14–16].

Although the natural products (1 and 2) do not show any significant phytotoxicity when tested on a number of specimens (Table 4) within the concentration limit studied, both of them (1 and 2) were found active against all the tested bacterial and fungal specimens. Compound (1) showed better antifungal as well as antibacterial activity in comparison to compound (2) (Table 1 and 2). However, both of them showed better activities against gram positive bacteria. Comparison amongst the gram negative bacteria revealed that compound 2 is more active. Both the observations are in accordance with the structure activity relationship as reported elsewhere[17–20]. Therefore, the out come of the investigation not only would enrich the understanding of structure and their biological activities among the lupane type of triterpenoid groups of natural products, but at the same time would provide a scientific base to the folk medicine culture in the tribal area.

**TABLE 4:** PHYTOTXICITY OF THE COMPOUNDS BASED ON THE LENGTH (IN CM) OF ROOTS AFTER 7 DAYS

Compounds	Concentration(μg/ml)	Rice	Wheat	Pea
Lupeol	Control	0.5	1.0	1.64
Betulinic acid	100	0.5	1.12	1.64
	250	0.5	1.12	1.67
	500	0.5	1.12	1.64
	100	0.5	1.21	1.56
	250	0.5	1.22	1.55
	500	0.5	1.25	1.56

Seeds of rice (*Oriza sativa*), wheat (*Triticum aestirium*), and pea (*Pisum sativum*) were collected from local market and used after washing.

## References

[1] Chhetri DR, Basnet D, Chiu PF, Kalikotay S, Chhetri G, Parajuli S (2005). Current status of ethnomedicinal plants in the Darjeeling Himalaya. Curr Sci.

[2] Bhattacharjee SK (1980). Chiranjeev banousadhi.

[3] Prajapati ND, Kumar U (2003). Agro’s dictionary of medicinal plants.

[4] Krishnamurti A (1969). The Wealth of India.

[5] Perry ML (1980). Medicinal Plants of East and south East Asia.

[6] Begum S, Hasan SI, Ali SN, Siddiqui BS (2004). Chemical Constituents from the Leaves of *Psidium guajava*. Nat Prod Res.

[7] Jairaj P, Khoohaswan P, Wongkrajang Y, Peungvicha P, Suriyawong P, Saraya ML (1999). Anti Cough and antimicrobial activities of *Psidium guajava* Linn. Leaf extract. J Ethnopharmacol.

[8] Jairaj P, Wongkrajang Y, Thongpraditchote S, Peungvicha P, Bunyapraphatsara N, Opartkiattikul N (2000). Guava leaf extract and topical haemostasis. Phytother Res.

[9] Lozoya X, Bercerril G, Martinez M (1990). Intraluminal perfusin model of *in vitro* guinea pig ileum as a model of study of the antidiarrheic properties of the Guava. Arch Invest Med (Mex).

[10] Qian H, Nohorimbere V (2004). Antioxidant Power of Phytochemicals from *Psidium guajava* Leaf. J Zhejiang Univ Sci.

[11] Abreu PR, Almeida MN, Bernardo RM, Bernardo LC, Garcia LC, Fonseen AS (2006). Guava extract (*Psidium guajava*) Alters the Labelling of Blood with technetium-99m. J Zhejiang Univ Sci B.

[12] Suleman P, Al-musallam A, Menezes CA (2002). The effect of biofungicide Mycostop on *Ceratocystis radicicola*, the causal agent of black scorch on date palm. Biol Control.

[13] Peng C, Bodenhausen G, Qiu SX, Fong HH, Farnsworth NR, Yuan SG (1998). Computer-assisted structure elucidation: Application of CISOC-SES to the resonance assignment and structure generation of betulinic acid. Magn Reson Chem.

[14] Sholichin M, Yamasaki K, Kasal R, Tanako O (1980). Carbon 13- nuclear magnetic resonance of lupine-type triterpene, lupeol, betulinic acid. Chem Pharm Bull.

[15] Gunasekera SP, Cordell GA, Farnsworth NR (1982). Constituents of *Pithecellobium multiflorum*. J Nat Prod.

[16] Garcia B, Alberto Macro J, Seonae E, Tortajada A (1981). Triterpenoids, waxes and tricin in *Phoenix canariensis*. J Nat Prod.

[17] Jigan P, Rathis N, Sumitra C (2005). Preliminary screening of some folklore medicinal plants from Western India for potential antimicrobial activity. Indian J Pharmacol.

[18] Recio MC, Giner RM, Máñez S, Ríos JL (1995). Structural requirements for the anti-inflammatory activity of natural triterpenoids. Planta Med.

[19] Walker R, Edward C (1999). Clinical pharmacy and therapeutics.

[20] Samania EF, Monache FD, Yunes RA, Paulert R, Samania A (2007). Antimicrobial activity of methyl australate from *Ganoderma australe*. Braz J Pharmacog.

